# First person – Shamsun Nahar

**DOI:** 10.1242/dmm.053037

**Published:** 2026-06-18

## Abstract

First Person is a series of interviews with the first authors of a selection of papers published in Disease Models & Mechanisms, helping researchers promote themselves alongside their papers. Shamsun Nahar is first author on ‘ Co-mutations of CTNNB1 and PTEN drive aggressive tumor progression in endometrial cancer’, published in DMM. Shamsun is in the lab of Jae-Wook Jeong at University of Missouri, investigating the molecular mechanisms underlying aggressive and metastatic endometrial cancer, with a focus on *Pten*/*Ctnnb1* co-mutations, EMT activation and therapeutic vulnerabilities by using genetically engineered mouse models and translational molecular approaches.

**Figure:**
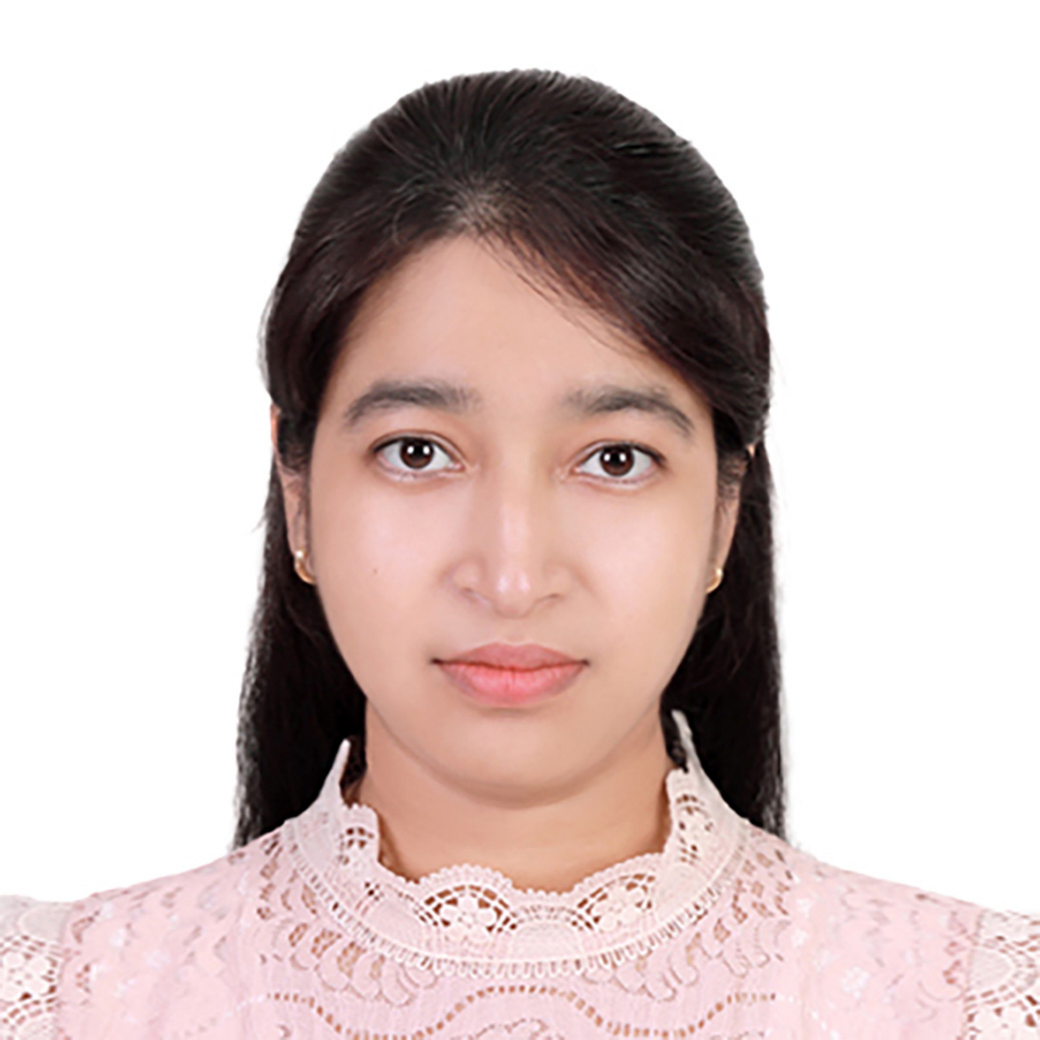
Shamsun Nahar

**Who or what inspired you to become a scientist?**

My mother always encouraged me to help others and make a positive impact on people's lives. As I grew older, I realized that science has the power to improve human health and contribute to society in meaningful ways, which inspired me deeply. During my undergraduate studies, I developed a strong interest in laboratory research and, over time, my curiosity and passion for scientific discovery continued to grow.

**What is the main question or challenge in disease biology you are addressing in this paper? How did you go about investigating your question or challenge?**

Previous studies in humans have shown that endometrial cancer together with mutation of β-catenin (CTNNB1) is associated with poor prognosis. To investigate the underlying mechanisms, we developed uterine-specific genetically engineered mouse models to examine how the oncogenic CTNNB1 cooperates with the commonly altered tumor suppressor PTEN in driving tumor progression. We aimed to determine how the combination of *Pten* loss and *Ctnnb1* activation promotes aggressive and metastatic endometrial cancer. To address this, we used these mouse models alongside histological analyses, fluorescent imaging, transcriptomic profiling and molecular studies to define how these co-mutations drive tumor progression, epithelial–mesenchymal transition (EMT), and invasion through activation of WNT/β-catenin and Hedgehog signaling pathways.

**How would you explain the main findings of your paper to non-scientific family and friends?**

We found that, when two important protective genes in the uterus stop working together properly the cancer grows much faster and becomes more aggressive. In our study, the cancer cells were able to spread into nearby tissues and other organs more easily. We also identified a possible therapeutic target in this process that may help researchers develop better treatments for aggressive endometrial cancer in the future.

Our study provides important insight into how specific genetic changes cooperate to drive aggressive and metastatic endometrial cancer.

**What are the potential implications of these results for disease biology and the possible impact on patients?**

Our study provides important insight into how specific genetic changes cooperate to drive aggressive and metastatic endometrial cancer. By uncovering the molecular mechanisms that promote early invasion and tumor spread, particularly through EMT and Hedgehog signaling, our findings identify potential therapeutic vulnerabilities in advanced-stage and poor-prognosis endometrial cancer. These results may help improve patient stratification, support the development of more targeted treatment strategies and, ultimately, contribute to better clinical outcomes for patients with aggressive endometrial tumors.

**Figure:**
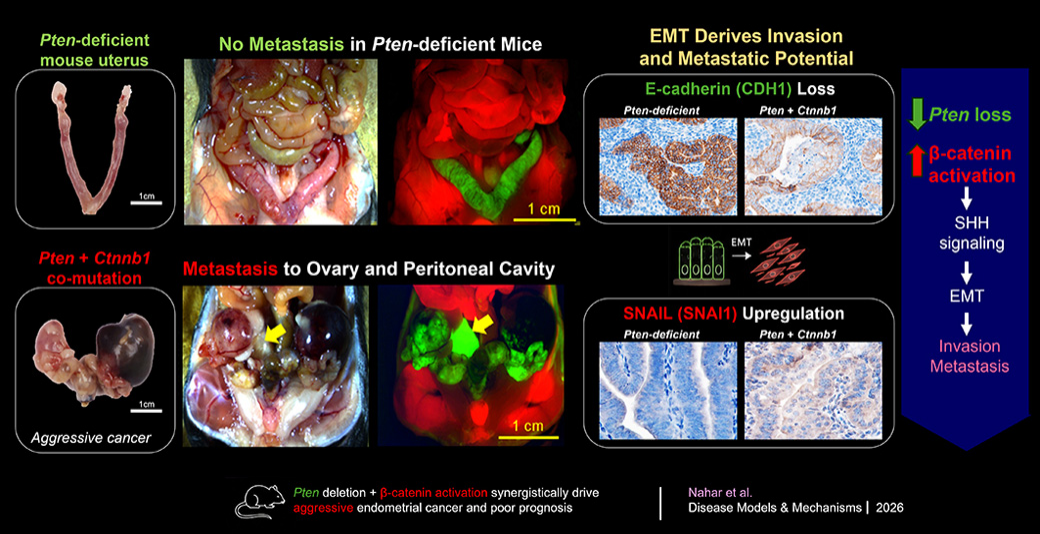
**PTEN and CTNNB1 co-mutations drive progression of aggressive endometrial cancer through EMT and metastatic dissemination.** Graphical summary illustrating the cooperative effects of uterine *Pten* loss and oncogenic *Ctnnb1* activation in promoting invasive endometrial cancer. Compared with PTEN-deficient uteri, co-mutant tumors exhibit enhanced tumor burden, metastatic spread, and activation of WNT/β-catenin and sonic hedgehog (SHH) signaling pathways. These molecular alterations promote epithelial–mesenchymal transition (EMT), characterized by loss of E-cadherin (CDH1) and upregulation of SNAIL (SNAI1), ultimately driving invasion and metastatic progression in a genetically engineered mouse model of endometrial cancer.

**Why did you choose DMM for your paper?**

We chose DMM because it is an excellent platform for studies that use disease models to uncover clinically relevant mechanisms. Our work combines a genetically engineered mouse model with translational cancer biology, which aligns well with the journal's focus and readership.

**Given your current role, what challenges do you face and what changes could improve the professional lives of other scientists in this role?**

One of the major challenges is balancing intensive experimental work with manuscript writing, data analysis and career development within limited time and funding. Greater access to stable research support, mentorship opportunities and resources for early-career scientists would help improve productivity, collaboration and long-term career growth.

**What's next for you?**

I plan to continue investigating the molecular mechanisms that drive aggressive and recurrent endometrial cancer, particularly, tumor microenvironment remodeling, metastasis and therapeutic vulnerabilities. My long-term goal is to contribute to the development of more effective targeted therapies for gynecologic cancers.

**Tell us something interesting about yourself that wouldn't be on your CV**

Outside the lab, one of the most meaningful parts of my life is spending time with my daughter Sia. Watching her grow reminds me of the importance of kindness, curiosity and compassion. I hope that, when she grows up, she will also strive to contribute positively to humanity and help others in her own way.

Animal models allow us to study how cancer develops and spreads in a complex biological environment that closely resembles human disease, helping us better understand mechanisms that cannot be captured in cell culture alone.

**Why are animal models important for studying cancer progression?**

Animal models allow us to study how cancer develops and spreads in a complex biological environment that closely resembles human disease, helping us better understand mechanisms that cannot be captured in cell culture alone.
